# Evidence and emerging trends in local therapy for metastatic hormone-sensitive prostate cancer: a narrative review

**DOI:** 10.1007/s12254-023-00934-w

**Published:** 2024-01-16

**Authors:** Stephan Brönimann, Nirmish Singla, Stephan M Korn, Nicolai A Huebner, Shahrokh F Shariat

**Affiliations:** Department of Urology, Comprehensive Cancer Center, Medical University of Vienna, Vienna, Austria; Department of Urology, The James Buchanan Brady Urological Institute, The Johns Hopkins University School of Medicine, Baltimore, MD, USA; Department of Urology, The James Buchanan Brady Urological Institute, The Johns Hopkins University School of Medicine, Baltimore, MD, USA; Department of Urology, Comprehensive Cancer Center, Medical University of Vienna, Vienna, Austria; Department of Urology, Comprehensive Cancer Center, Medical University of Vienna, Vienna, Austria; Department of Urology, Comprehensive Cancer Center, Medical University of Vienna, Vienna, Austria; Hourani Center for Applied Scientific Research, Al-Ahliyya Amman University, Amman, Jordan; Karl Landsteiner Institute of Urology and Andrology, Vienna, Austria; Department of Urology, Weill Cornell Medical College, New York, NY, USA; Department of Urology, University of Texas Southwestern, Dallas, TX, USA; Department of Urology, Second Faculty of Medicine, Charles University, Prague, Czech Republic

**Keywords:** Prostate cancer, Oligometastatic, Local therapy, Cytoreductive prostatectomy, Radiation therapy

## Abstract

**Introduction:**

Metastatic hormone-sensitive prostate cancer (mHSPC) displays both simultaneous and sequential patterns of metastasis, emphasizing a comprehensive treatment approach that integrates both local therapy and systemic treatment strategies. The increasing use of molecular imaging has led to a rise in mHSPC diagnoses, underscoring the importance of identifying the right patient population and effective treatment concepts for this disease state.

**Results:**

Two prospective trials, HORRAD and STAMP EDE, investigated prostate radiotherapy (RT) for mHSPC; however, they did not show an overall survival (OS) benefit in the unselected cohort. Nonetheless, RT showed favorable outcomes in patients with fewer than five bone metastases, resulting in a 7% 3-year survival improvement and supporting the integration of RT in multimodal treatment for men with oligometastatic mHSPC. Regarding cytoreductive prostatectomy (cRP), the TRoMbone Trial confirmed its feasibility and safety. In addition, findings from the FUSCC-OMPCa Trial demonstrated improved 3-year radiographic progression-free survival and OS rates with acceptable rates of complications and incontinence. Recent data from the LoMP registry have further supported superior OS and cancer-specific survival (CSS) in patients undergoing cRP compared to systemic therapy alone. Notably, no significant differences in OS and CSS were observed between the cRP and RT groups. However, cRP-treated patients exhibited superior 2-year local event-free survival when compared to those treated with RT.

**Conclusion:**

RT in combination with systemic therapy remains the established first-line treatment for low-burden mHSPC, though the exact definition of low metastatic burden remains contentious. Precise assessment of metastatic burden is vital to identify patients who would derive the greatest benefit from RT. As treatment paradigms evolve, embracing multimodal approaches holds potential for optimizing outcomes in patients with mHSPC. Further research is needed to solidify the role of cRP as a standard therapeutic approach and to refine treatment strategies for improved patient outcomes.

## Introduction

In the context of prostate cancer, development of metastatic lesions can be delineated into two principal pathways: one emanating directly from the primary tumor and the other emanating from metastatic sites. The inherent nature of this disease characteristic unveils a promising treatment strategy, wherein the primary tumor can be effectively targeted through a combination of local therapeutic and systemic treatment options. This approach forms a comprehensive multimodal treatment strategy for men diagnosed with metastatic hormone-sensitive prostate cancer (mHSPC) [1]. Furthermore, the implementation of novel molecular imaging techniques has resulted in a surge in the diagnosis of mHSPC cases [2]. Given the significant increase in diagnoses, there is a pressing and unaddressed necessity to determine the best-suited multimodal treatment for individuals with limited metastatic burden. It is paramount to tailor treatment modalities to each patient, in alignment with the principles of personalized medicine, to mitigate the risk of both over- and undertreatment. Randomized controlled trials evaluated radiation therapy (RT) in men with mHSPC; however, evidence for cytoreductive radical prostatectomy (cRP) remains scarce. In this review, we will delve into these concepts, discussing the current state of local therapy options for men with mHSPC. By evaluating currently available clinical trials, we aim to provide a concise yet comprehensive overview of optimal management strategies for patients diagnosed with mHSPC.

## Results

### Radiation therapy

Two prospective trials have evaluated the impact of prostate radiotherapy (RT) in the context of metastatic hormone-sensitive prostate cancer (mHSPC) [3, 4]. The HORRAD trial enrolled 432 patients diagnosed with mHSPC, who were randomly assigned to receive either androgen deprivation therapy (ADT) alone or a combination of ADT with intensity-modulated radiotherapy (IMRT) employing image-guided radiotherapy (IGRT) targeting the prostate. The findings unveiled that the addition of RT significantly extended the median time to prostate-specific antigen (PSA) progression (hazard ratio [HR] 0.78, 95% confidence interval [CI] 0.63–0.97). However, no substantial difference in overall survival (OS) was observed between the treatment arms (HR 0.9, 95% CI 0.7–1.14) [3].

In the STAMPEDE trial, which included 2061 men with mHSPC, participants were randomly assigned to receive either ADT alone or ADT in combination with prostate RT. The study revealed a noteworthy distinction based on metastatic burden. Specifically, prostate RT exhibited a significant improvement in OS for patients with low metastatic burden (202 deaths in the ADT-alone group versus 156 deaths in the ADT+RT group; HR 0.64, 95% CI 0.52–0.79, *p*<0.001). However, in patients with high metastatic burden according to the CHAARTED definition [5], the difference in OS was not statistically significant (375 deaths in the ADT-alone group versus 386 deaths in the ADT+RT group; HR 1.11, 95% CI 0.96–1.28, *p*=0.164). The interaction analysis demonstrated a significant distinction between the two groups (interaction *p*<0.001) [4].

In a comprehensive meta-analysis that encompassed data from both the HORRAD and STAMPEDE trials, which involved a total of 2126 men with 969 deaths, no survival benefit was observed in the unselected cohort. The analysis yielded no supportive evidence for the use of prostate RT in terms of OS (HR 0.92, 95% CI 0.81–1.04, *p*=0.195) or progression-free survival (PFS, HR 0.94, 95% CI 0.84–1.05, *p*= 0.238). However, a remarkable and statistically significant improvement was observed in terms of biochemical progression (HR 0.74, 95% CI 0.67–0.82, *p*= 0.94×10^−8^) and freedom from failure (FFS, HR 0.76, 95% CI 0.69–0.84, *p*=0.64×10^−7^), leading to a 10% benefit at 3 years. In addition, a notable distinction emerged in the impact of metastatic burden on survival, particularly for men with four or fewer bone metastases (HR 0.73, 95% CI 0.58–0.92, *p*=0.0071), leading to an absolute improvement of 7% in 3-year survival (from 70 to 77%). Conversely, there was insufficient evidence of a substantial benefit among men with five or more bone metastases (HR 1.07, 95% CI 0.92–1.26, *p*=0.37). Similar trends were observed in PFS (interaction HR 1.32, 95% CI 1.04–1.67, *p*=0.021) and freedom from failure (FFS; interaction HR 1.35, 95% CI 1.10–1.66, *p*=0.004) [6].

Taken together, those two randomized clinical trials, HORRAD and STAMPEDE, have provided strong evidence supporting the use of RT to improve survival outcomes in individuals diagnosed with low-metastatic-burden prostate cancer. Consequently, there is widespread agreement to include prostate RT as a standard first-line treatment option for men with newly diagnosed, low-metastatic-burden disease. However, there remains a contentious issue concerning the specific definition of low metastatic burden. Existing criteria dichotomize patients into low-burden or high-burden subgroups based on different thresholds of bone metastasis counts, which have proven prognostic significance in patients receiving systemic therapy for prostate cancer [7, 8]. As a result, the optimal threshold for bone metastatic burden, indicating potential benefits of prostate RT in men newly diagnosed with mHSPC, has not been systematically assessed, especially taking into account that both studies used conventional imaging to define metastatic burden. To address the dichotomous definition, a secondary analysis of the STAMPEDE trial revealed compelling evidence indicating the benefit of prostate radiotherapy in patients with ≤3 bone metastases and M1a disease [9]. In this subgroup analysis, which included a total of 1939 out of 2061 men, a clear association was observed between bone metastasis counts and the benefits of prostate RT on OS and FFS, which decreased continuously as the number of bone metastases increased, with benefit most pronounced for up to 3 bone metastases. Upon further analysis of subgroups, it was observed that the addition of prostate RT resulted in a greater degree of benefit for patients with low metastatic burden, specifically those who had only nonregional lymph nodes (M1a) or 3 or fewer bone metastases without visceral metastasis. In this subgroup, the HR for OS were 0.62 (95% CI 0.46–0.83), and for FFS, the HR was 0.57 (95% CI 0.47–0.70). However, in patients with 4 or more bone metastases or any visceral/other metastasis, the magnitude of benefit was diminished, as evidenced by HRs for OS of 1.08 (95% CI 0.91–1.28; interaction *p*=0.003) and for FFS of 0.87 (95% CI 0.76–0.99; interaction *p*=0.002) [9].

In terms of side effects, the administration of prostate RT resulted in severe acute bladder toxicity for 4% of men, and 1% experienced severe acute bowel toxicity, according to the RTOG scale. The reported outcomes from the STAMPEDE trial indicated that 4% of men encountered severe late effects. However, no significant difference was found in Global Quality of Life (QoL) or QLQ-30 Summary Score between the groups. Interestingly, no evidence indicating a significant difference in time to symptomatic local events was found [4].

In summary, the evidence from randomized prospective trials highlights the benefits of radiotherapy for men with low metastatic burden, particularly in terms of OS and FFS. However, it is important to note that no significant differences were observed in the timing of symptomatic local events, prompting the need for more comprehensive scientific investigations to explore the potential role of surgical interventions within this specific disease context.

### Cytoreductive prostatectomy

While RT combined with systemic therapy has become the standard of care in patients with low-volume mHSPC discovered on conventional imaging, cytoreductive radical prostatectomy (cRP) remains an experimental approach, with the goal of minimizing complications from local progression while maintaining the oncologic benefit of local therapy. The evidence for cRP, despite being highly promising in terms of oncologic outcomes, local control, and favorable quality of life (QoL), has mostly been shown in small retrospective series, which often suffer from selection bias (e.g. highly selected patients with no comorbidities, healthier CRP group, less metastatic burden in the CRP group [10]).

The TRoMbone trial, a randomized controlled trial (RCT), investigated the feasibility and safety of cRP for patients with newly diagnosed oligometastatic mHSPC in comparison to ADT alone or in combination. The trial findings indicated that cRP was found to be feasible and safe, with no significant impact on QoL. However, the reported outcomes focused primarily on early oncologic measures, such as positive surgical margins (PSM) and postoperative PSA levels. Further evaluation of long-term oncologic outcomes is, therefore, warranted [11].

The FUSCC-OMPCa trial, a prospective phase 2 RCT, aimed to assess the potential survival benefits of radical local therapy (RLT) in newly diagnosed oligometastatic mHSPC patients [12]. In this study, a total of 200 patients were randomly assigned to receive ADT alone or ADT combined with RLT, with the latter primarily involving cRP in 85% of cases. Following cRP, 42% of patients had PSM, and at 6 weeks post-surgery, 66% of patients achieved a PSA level below 0.2ng/ml. Over a median follow-up period of 4 years, the addition of RLT to ADT resulted in a notable reduction in the risk of radiographic progression by 57% (HR 0.43, 95% CI 0.27–0.70, *p*=0.001) and a 56% decrease in the risk of death (HR 0.44, 95% CI 0.24–0.81, *p*=0.008).

In two recent publications [13, 14], the outcomes of the prospective multicenter Belgian Local Treatment of Metastatic Prostate Cancer (LoMP) registry were published, encompassing patients with mHSPC subjected to local therapy, involving either cRP or RT. Lumen et al. [13] reported that over a median follow-up of 32 months, the cRP group (*n*=48) exhibited superior 2-year OS and cancer-specific survival (CSS) rates compared to the group receiving systemic therapy alone (93% vs 69% and 93% vs 75%, respectively). It should be noted that the study’s non-randomized, observational, and patient-preference design led to the cRP arm comprising younger men with lower PSA levels, more frequent organ-confined disease, and better performance status than those in the systemic therapy arm. Interestingly, no significant differences in OS and CSS were evident between the cRP and RT groups, yet cRP-treated patients demonstrated better 2-year local event-free survival in comparison to their RT counterparts (92% vs 77%). Another study based on the same database, with a median follow-up of 35 months (interquartile range 24–47), revealed that the 3-year castration-resistant prostate cancer (CRPC)-free survival was significantly higher in the cRP group (*n*=40) in comparison to the ADT-alone group (59% vs 40%, *p*=0.02). Nevertheless, upon conducting a multivariable analysis with propensity-score matching, cRP did not emerge as an independent factor for CRPC-free survival (HR 0.93, 95% CI 0.37–2.38, *p*=0.9) [14].

Regarding functional outcomes, the FUSCC-OMPCa trial provided valuable insights, with 24 patients (28%) experiencing perioperative complications within 90 days after cRP, 3 of whom (3.5% of all) exhibited Clavien–Dindo grade (CDG) >3a complications, encompassing pelvic hematoma, anastomotic stenosis, and rectal injury. Notably, 8% of patients encountered urinary incontinence during the first-year post-surgery, which subsequently decreased to 5% during the second year. Furthermore, analysis using the European Organisation for Research and Treatment of Cancer (EORTC) QLQ-PR25 questionnaire unveiled that long-term grade 3–4 side effects, such as micturition difficulties, urgent urination, odynuria, urinary incontinence, hot flashes, and breast distending pain, were evident in less than 13% of patients [12]. Additionally, the aforementioned LoMP registry also investigated the incidence of local events (LE) necessitating invasive treatment, including catheterization or any invasive procedure involving the urinary tract, as potential complications following cRP or RT subsequent to ADT initiation [13]. A significant reduction in LE risk and an increase in 2-year local event-free survival were discerned in the cRP group compared to individuals subjected to RT and systemic therapy alone [13]. Buelens et al. reported that CDG <3 complications were observed within 3 months post-cRP in 18 patients (45%). Remarkably, no CDG 4 or 5 complications were encountered. Among patients who underwent cRP, 31 individuals (79%) exhibited continence one year after the procedure. During the follow-up, urinary obstruction was observed, necessitating additional surgery to address bladder outlet obstruction and ureteric stricture in 7.5 and 5.0% of patients, respectively. In contrast, a significantly higher proportion of patients (38 and 2.5%, respectively) treated with standard of care experienced such complications [14].

Limited prospective evidence suggests that cRP may be associated with favorable oncologic outcomes when compared with systemic therapy alone in the context of low-volume mHSPC. However, the success of cRP relies heavily on appropriate patient selection, which plays a crucial role in achieving optimal short- and medium-term outcomes. Encouragingly, available prospective studies have highlighted the positive impact of cRP on local disease control, demonstrating a significant reduction in adverse events and acceptable rates of complications and incontinence. Despite these promising findings, it is important to acknowledge the current limitations in the research, particularly the absence of phase 3 RCTs investigating the effectiveness of cRP. Furthermore, the interpretation of results from existing and ongoing studies poses challenges. The inclusion criteria for clinical trials on cRP vary with regard to the extent of metastases and T-stages. Moreover, they also differ with regard of the used imaging modality for staging. Standardizing imaging modalities in these studies is crucial, as significant variations in metastasis detection rates can lead to the Will Rogers effect, causing data discrepancies [15]. In addition, it is worth emphasizing that the “best systemic therapy” or “standard systemic therapy” has already or will probably evolve over time, while these studies are enrolling participants or patients are still in the follow-up period. Finally, as those studies also use differing primary and secondary endpoints, those need to be thoroughly understood to prevent confusion and ensure meaningful comparisons among ongoing studies, thereby enabling correct contextualization of their findings [15].

Consequently, cRP remains an investigational therapeutic approach, necessitating further comprehensive research before its widespread adoption in the management of patients with low-volume mHSPC.

## Conclusion

The combination of prostate radiotherapy (RT) and systemic therapy should be offered to men with newly diagnosed, low-burden mHSPC. The HORRAD and STAMPEDE trials have provided strong evidence supporting the use of prostate RT in improving survival in this patient population [3, 4]. The definition of low metastatic burden in prostate cancer remains a subject of divergence, leading to uncertainty and debate regarding the precise criteria for patient selection in clinical practice. According to the available evidence, the benefits of prostate RT are most pronounced in patients with up to 5 bone metastases on conventional imaging, leading to an absolute improvement of 7% in 3-year survival (from 70 to 77%) [4], or as shown in a secondary analysis of the STAMPEDE trial, an even more pronounced benefit in patients with up to 3 bone metastases [9]. It is essential to accurately assess the metastatic burden to identify those who would benefit most from prostate RT. While newer imaging techniques like prostate-specific membrane antigen positron emission tomography (PSMA PET) and whole-body magnetic resonance imaging (MRI) are not yet included in the definition of metastatic disease burden, they hold promise for further refining patient selection in the future.

Given the constantly evolving treatment landscape in prostate cancer management, it is becoming increasingly evident that a multimodal approach holds promise for achieving optimal outcomes in patients with low-volume metastatic hormone-sensitive prostate cancer (mHSPC). As treatment paradigms continue to evolve, it should be noted that not only imaging techniques, but also the landscape of systemic therapy for metastatic prostate cancer has also changed, since the trials were conducted [16]. The addition of new hormone agents, such as abiraterone, apalutamide, or enzalutamide in addition to androgen deprivation therapy (ADT) has become a standard approach for low-burden metastatic disease [16]. Nevertheless, the intricate interplay between RT and the new hormonal agents in terms of their combined effects on survival benefits in patients with prostate cancer is yet to be comprehensively elucidated.

Regarding cytoreductive prostatectomy (cRP), despite showing feasibility, promising oncologic outcomes and favorable quality of life, there is a lack of phase 3 randomized clinical trials supporting its broad adoption for low-volume mHSPC. Further research is needed to establish its role as a standard therapeutic approach.

As of now, it is reasonable to consider prostate RT and one of the new hormonal agents as part of the standard of care for low-volume mHSPC alongside ADT. However, ongoing research and emerging evidence may lead to continuous refinements in the guidelines, offering more personalized and effective treatment options. The aim is to maximize disease control, improve survival rates, and enhance the overall quality of life for these patients.

As we gather more data and gain deeper insights into the intricate biology and behavior of low-volume mHSPC, treatment protocols are likely to evolve. Multidisciplinary approaches and precision medicine may hold the key to tailoring therapies based on individual patient characteristics, disease burden, and response to treatment. It is essential for clinicians and researchers to collaborate, pool their expertise, and conduct rigorous clinical trials to generate high-quality evidence, thereby paving the way for evidence-based treatment decisions. With the continuous advancement of medical knowledge and therapeutic innovations, the future of managing low-volume mHSPC appears promising, with the potential for significant improvements in patient outcomes.
